# Data on foliar nutrient concentration of invasive plants in the recipient habitat and their native habitat

**DOI:** 10.1016/j.dib.2019.104201

**Published:** 2019-06-27

**Authors:** Pushpa Soti, Matthew F. Purcell, Krish Jayachandran

**Affiliations:** a University of Texas Rio Grande Valley, USA; b USDA ARS Australian Biological Control Laboratory, Australia; c Florida International University, USA

**Keywords:** Exotic invasive species, Plant growth

## Abstract

Higher foliar nitrogen concentration in plants is often attributed to higher biomass assimilation and subsequently higher plant growth rate. To understand the underlying mechanism of extensive growth rate of an invasive plant, Old World climbing fern (*Lygodium microphyllum),* we analyzed the leaf tissue samples from the native and invaded habitats. In each habitat we selected 3 different locations with varying habitat characteristics (soil type, land use history and coexisting vegetation). Plant aboveground tissue collected from each site were analyzed for macro and micro nutrients. Total C and N were measured with a Truspec CN Analyzer. Total Ca, Fe, Mg, K, Mn, and P in plant tissue samples were measured using inductively coupled plasma mass spectrometry (ICP –MS). Here we present the difference in foliar nutrient concentration of invasive plant species in their native habitats and invaded habitats.

**Table:** Specifications Table

Subject area	Ecology
More specific subject area	Invasion ecology, plant sciences
Type of data	Map, table and pictures
How data was acquired	Field survey and lab analysis using ICP –MS, Truspec CN analyzer
Data format	Raw and analyzed
Experimental factors	Fully grown young plant tissue were collected for nutrient analysis from all the sampling sites.
Experimental features	Data were subjected to analysis of variance (ANOVA) using SAS Version 9.2 software, and means were separated using Fisher LSD (*P*-values ≤ 0.05).
Data source location	Australia (Native)
	Site 1.16°15′25.57″S, 145°24′3.94″E
	Site 2.27°40′4.16″S, 153°16′0.44″E
	Site 3.27°22′31.12″S, 153° 5′39.42″E
	Florida (Recipient)
	Site 1.26° 4′0.04″N, 80° 16′ 5.88″W
	Site 2.28° 23′ 4.03″ N, 81° 44′ 41.30″ W
	Site 3.27°0′37.33″N, 80°7′20.28″W
Data accessibility	Data are available within this article.
Related research article	Soti, P. G., Jayachandran, K., Purcell, M., Volin, J. C., & Kitajima, K. (2014). Mycorrhizal symbiosis and *Lygodium microphyllum* invasion in south Florida—a biogeographic comparison. *Symbiosis*, *62* (2), 81–90 [1].

**Table:** 

**Value of the Data** • To our knowledge this is the first comparative data on foliar nutrients concentration of an invasive plants growing in their native habitats and in the invaded or recipient habitats. • This dataset can potentially provide some insight on the extensive aboveground growth and nutrient turnover rate of an invasive species in the recipient habitats. • This data can be useful to researchers studying the ecology of exotic invasive plants.

## 1 Data

**Fig. 1:**
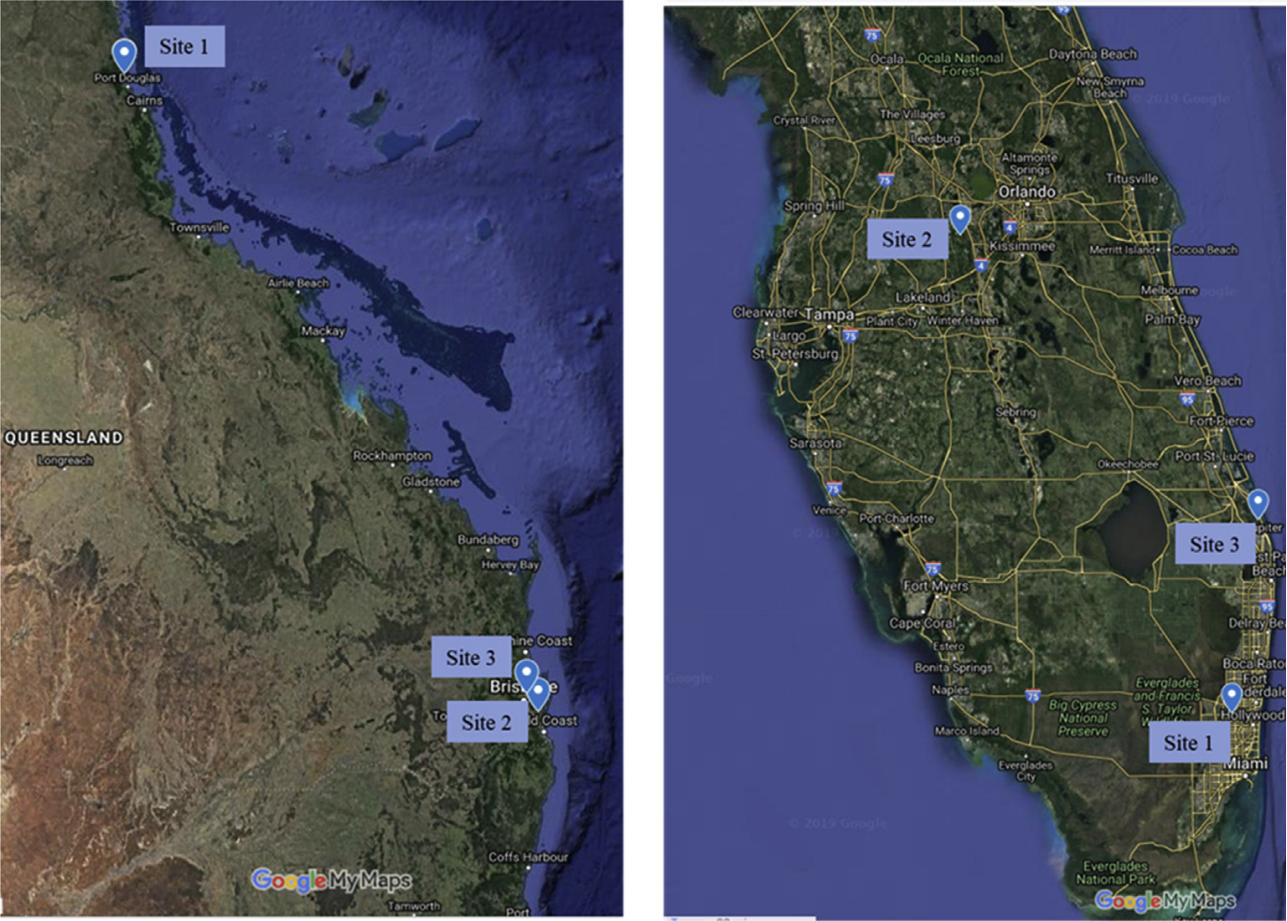
Sampling sites in native habitat, Queensland Australia (left) and recipient habitat, Florida (right).

**Fig. 2:**
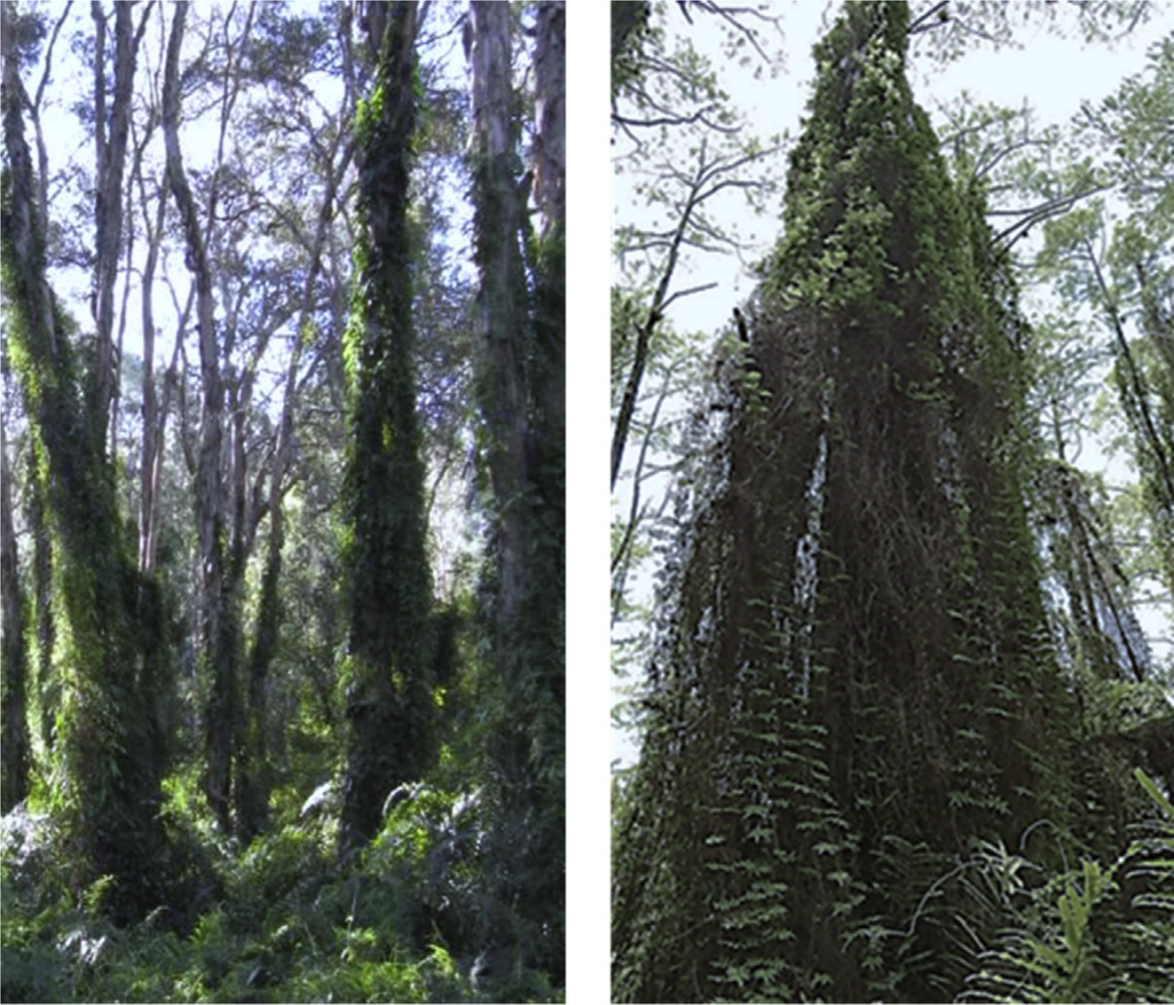
Above ground growth of *L. microphyllum* in native habitat (left) and recipient habitat (right). In the recipient habitats in Florida, *L. microphyllum* grows over trees up to 30 m in height and creates thick fern mats, smothering trees and shrubs, however in their native habitats these plants are much smaller in height and do not create a thick fern mat.

We present the data collected from an extensive survey of a highly invasive plant, *Lygodium microphyllum*, in its native habitat in Queensland, Australia and recipient habitat in Florida, United States (Fig. 1). The difference in above ground growth of *L. microphyllum* in both the habitats is presented in Fig. 2. Data on the variation in the plant tissue nutrient content is presented in Table 1.

**Table 1:** Nutrient concentration in the leaf tissue from the two habitats (recipient and native) of *L. microphyllum*. Different letters within the column indicate significantly different means at the 0.05 level.

	Ca (mg g^−1^)	Fe (mg g^−1^)	Mg (mg g^−1^)	K (mg g^−1^)	Mn (mg g^−1^)	Zn (mg g^−1^)	C:N
Recipient							
Site 1	5.73b	0.22a	2.28ab	23.54ab	0.12a	0.10a	12.70c
Site 2	7.37a	0.14b	1.99bc	20.49bc	0.10b	0.09ab	16.11b
Site 3	5.11b	0.15b	2.55a	18.97c	0.09b	0.06c	12.33c
Native							
Site 1	3.24cd	0.12b	1.68c	18.10c	0.08c	0.07bc	25.00a
Site 2	2.82d	0.12b	1.83c	24.94a	0.06d	0.10a	23.738a
Site 3	3.97c	0.14b	1.73c	19.63c	0.08c	0.08abc	12.33a

## 2 Experimental design, materials, and methods

### 2.1 Sampling sites

Leaf tissue samples of wild *L. microphyllum* were collected from 3 different locations each in south Florida and Queensland, Australia (Fig. 1). In each location young, fully-grown aboveground plant tissue samples were collected from 6 different plants selected randomly resulting a total of 36 samples.

### 2.2 Sample processing and analysis

The plant tissue samples were dried in an oven at 60 °C for one week and finely ground using a mortar and pestle. Total C and N were measured with a Truspec CN analyzer. Total Ca, Fe, Mg, K, Mn, and P in plant tissue samples were measured with an ICP –MS at USDA, ARS Laboratory, Miami, Florida.

Samples for ICP-MS analysis were prepared following the slightly modified acid digestion method [2]. 0.5 g of finely ground plant tissue samples were transferred to large glass tubes and mixed with 10 ml of 30% HNO_3_. The tubes were covered with a vapor recovery system and heated to 95±5 °C and refluxed for 10 minutes without boiling under the hood in a heating block maintained with a Partlow Mic 6000 Profile Process Controller. After cooling to 40 °C, 2 ml of DI water and 3 ml of 30% H_2_O_2_ was added and heated until the effervescence subsided. The samples were cooled and diluted to 50 ml with DI water, centrifuged at 2000 rpm for 10 minutes and filtered with a Whatman No. 41 filter paper.

### 2.3 Data analysis

Data on the foliar nutrient concentration were subjected to analysis of variance (ANOVA) using SAS Version 9.2 software. Means were separated using Fisher LSD (*P*-values ≤ 0.05).
